# Quality of life considerations in head and neck cancer: United Kingdom National Multidisciplinary Guidelines

**DOI:** 10.1017/S0022215116000438

**Published:** 2016-05

**Authors:** S N Rogers, C Semple, M Babb, G Humphris

**Affiliations:** 1 Evidence-Based Practice Research Centre, Faculty of Health, Edge Hill University, Ormskirk, UK; 2 Regional Maxillofacial Unit, Aintree University Hospitals NHS Foundation Trust, Liverpool, UK; 3 Department of Head and Neck Cancer, Cancer Services, South Eastern Health & Social Care Trust, Belfast, UK; 4 NCRI Head and Neck Clinical Studies Group, NCIN Head and Neck Site Specific Clinical Reference Group, B16 – Complex Head and Neck Clinical Reference Group, Chesterfield, Derbyshire, UK; 5 Medical School, University of St Andrews, St Andrews, UK

## Abstract

This is the official guideline endorsed by the specialty associations involved in the care of head and neck cancer patients in the UK. It identifies the current evidence base and role of health-related quality of life assessment for this group of patients.

**Recommendations:**

• Health-related quality of life is integral to treatment planning, refining treatment protocols, and more personalised follow-up support. (G)

• Health-related quality of life and patient concerns should be regularly assessed during patient care. (G)

• Health-related quality of life assessment and patient concerns on an individual patient basis can be helpful to trigger multi-professional support and interventions. (G)

The evaluation of the quality of life (QoL) in patients with head and neck cancer is integral to optimal patient care.^1^ Survival is usually the initial primary concern of patients and the focus is on treatments that offer the best chance of cure as a priority. However, after treatment there tends to be a shift towards QoL and living with the consequences of head and neck cancer treatment (survivorship).

## What is quality of life?

Quality of life is a multifaceted construct comprising many different aspects leading to numerous definitions. The World Health Organization defines quality of life as an “*individual*'*s perception of their position in life in the context of the culture and value systems in which they live and in relation to their goals, expectations, standards and concerns*”.^2^ Quality of life comprises a person's physical health and functioning, psychological state, level of independence, social relationships, occupation and finance, and personal beliefs. There is a complex relationship between factors such as the characteristics of the individual with respect to symptoms, personality, motivation, value preferences and the characteristics of the environment such as psychological, social and economic support.^2^ The term ‘health-related quality of life’ (HRQoL) is more disease specific and allows the healthcare professions to focus upon the assessment of the impact of the disease and its treatment on the physical, psychological and social aspects.^3^

## Why should we measure quality of life?

Health-related quality of life evaluation gives an indication of how the patient perceives the impact of their cancer and its treatment. This information can be used to give the patient and their family an indication of ‘what will I be like’.^1^ This patient reported outcome allows the health professional an opportunity to reflect on the patient's reaction. Individual patient-rated outcomes can often differ quite markedly from clinician-rated scores. Health-related quality of life measurement has a role in evaluating treatment outcomes, helping to define treatment protocols, as primary or secondary outcome(s) of clinical trials, providing additional information to assist in individual decision-making processes, to support the identification of poor outcomes, so that intervention and support can be considered.^4^ Checklists such as the Patients Concerns Inventory help patients express unmet concerns and can be used as part of holistic needs assessment.^5^ A better understanding of patients' perception helps facilitate improvements in aftercare and serves to drive clinically relevant outcomes research.^6^ Also patient-reported outcomes should be part of national outcome datasets.^7^^–^^9^

It is appreciated that there are many potential difficulties in assessing HRQoL in clinical practice.^10^^,^^11^ Perhaps the biggest challenges are: (i) the burden of administration and processing of the questionnaires; (ii) the reality that patients tend to adapt over time, so that expected differences between treatments might not be as significant as anticipated; (iii) that HRQoL data are weighted to survivors; and (iv) that there is little evidence of agreed standards of analysis and reporting. Another barrier is the lack of evidence as to when HRQoL should have a major role on treatment decisions, or an important role simply as an additional factor, or perhaps where it has relatively little value. Hence, healthcare professionals can unrealistically rely too much on the value of HRQoL in certain clinical situations and this can lead to frustration and a perceived lack of benefit in the HRQoL process.

## How should it be measured?

The commonest way to measure HRQoL is by patient self-completed questionnaire (quantitative) although other methods include open and semi-structured interview (qualitative).^11^ There is no gold standard questionnaire and each has its own unique features and merits.^12^^–^^14^ All questionnaires are inherently limited by the range of issues addressed, the wording used, and the scoring systems. The choice of questionnaire depends on the reason for using it, e.g. research, audit, integrated into routine clinical practice or to assist in the evaluation of a specific functional outcome.^15^

Questionnaires can be used either cross-sectionally or longitudinally. Longitudinal data from pre-treatment has the distinct advantage of allowing the measurement of change and also recording HRQoL during the different phases of treatment. It is a logistical challenge to ensure patients self-complete questionnaires before treatment and at regular intervals subsequently. Cross-sectional evaluation is simpler to conduct and easier to achieve larger patient numbers when stratifying for patient characteristics. Questionnaires can be divided into four main categories: (i) those asking on a range of broad issues not specific to cancer; (ii) those addressing issues common to all cancers; (iii) questionnaires with items specific to head and neck cancer; and (iv) those questionnaires that focus in detail on a particular aspect of head and neck function.^9^

With changes in treatments e.g. epidermal growth factor receptor inhibitors as part of chemotherapy, so existing HRQoL questionnaires might need to be modified to include additional side effects and functional deficits. As the relationship between unmet need and HRQoL becomes more clearly understood, further consideration needs to be given as to how, within the financial constraints of cancer care, questionnaires can be more easily integrated into routine practice. Advances in technology will assist in the collection and inclusion of patient-reported outcomes. The almost ubiquitous ownership of mobile phones allows developers in partnership with clinical researchers to construct ‘Apps’ that can send alerts to patients for HRQoL updates on certain features. This is an exciting area that is in its infancy but holds great promise to enable a more comprehensive, flexible and frequent opportunity to explore, study and intervene in patient HRQoL.

## What are the key issues?

There are a considerable range of issues that impact on the HRQoL outcomes following head and neck cancer. This section makes only very brief comment on the type of issues involved (listed in alphabetical order). There are several review articles that give additional information.^12^^–^^14^^,^^16^^,^^17^ At the present time there tends to be a lack of long-term outcomes reported in the literature. Also newer treatment strategies are under reported given the time necessary to get adequate HRQoL information.

• Carer: there is a need to promote positive carer support; carers can underestimate the HRQoL outcome
• Comorbidity: patient perception of disability, rather than the extent and severity of disease is of major influence in head and neck HRQoL
• Coping: social support seeking is beneficial whilst avoidance is bad
• Dental status: eating – social interaction and is linked to coping
• Disfigurement: appearance, body image, not only an issue in surgical patients
• Emotion: anxiety is high pre-treatment; mood disturbance and/or depression is treatable
• Family and children: the impact of cancer affects family and community
• Fatigue: common in the first year post-treatment; poor sleep; low energy
• Fear of recurrence: unpredictable by clinical characteristics; does not lessen over time; and high levels predict higher consumption of formal healthcare.
• Financial and work: employment; benefits; cost of treatment and follow-up; and retirement
• Function: pre-existing comorbidities; problems of combination treatment modalities – impact on recreation, hobbies, interests. In general, the less the consequence of the cancer and its treatment in terms of social function the better the HRQoL outcomes
• Fungating wounds: difficulties in palliation in head and neck; relatively few published papers
• Information: varying amounts, in various ways, at different times the importance of communication skills and consistency of contact with named health professional for duration of clinical treatment; also access to patient and career support groups
• Intimacy: sexuality, worst in the younger patient as an unmet need
• Lifestyle choices: smoking; alcohol abuse
• Nutrition: low weight; diet; gastrostomy feeding
• Oral rehabilitation: chewing and/or eating – realistic expectations of rehabilitation
• Osteoradionecrosis: associated with pain; trismus; poor HRQoL; and nutrition problems
• Pain: need for opiates; poor sleep; linked with depression
• Personality: optimism and HRQoL and survival; high neuroticism poor HRQoL
• Self-esteem: social concerns; reactions of friends, wider community, work colleague; low self-esteem associated with poor HRQoL
• Sociodemographic: deprivation and social support; age; and finance
• Speech: complex function; various aspects; laryngeal speech outcomes; isolation
• Swallowing: nutrition; social; presence of feeding tube most significant to HRQOL
• Shoulder: shoulder discomfort and neck tightness; debate around avoiding a neck dissection or carrying out a selective dissection
• Trismus: difficulty in mouth opening associated with diet, social, and dental health
• Unknown: clinical art of the individual patient not a precise science
• Xerostomia: dry mouth has a profound impact on social function and HRQoL, intensity modulated radiation therapy should be used whenever feasible.

## Examples of how HRQoL might change practice

Health-related quality of life is a factor that is weighed against treatment burden and toxicity, and also any survival benefit between treatments. In the three common head and neck cancer sites, HRQoL might be a driver for evolving strategies alongside other drivers such as survival, function and healthcare cost. Examples are described below.

### Oropharynx

1. Early stage disease: There is an argument for transoral excision for early oropharynx lesions with selective neck dissection. This avoids the need for free tissue transfer and access procedures such as lip split mandibulotomy.
  *Drivers for change*: Health-related quality of life, survival, function, cost to National Health Service (reduced length of stay).
2. Advanced stage disease: Chemoradiotherapy is often advocated for larger oropharyngeal primaries if laser resection is not possible. The long-term outcomes remain unclear as does the success of salvage surgery and its impact on HRQoL. The benefit of salvage surgery and the impact on HRQoL is currently unclear. Transoral surgery is problematic due to the high-risk of local necrosis, non-healing and catastrophic bleeding. The use of free flap reconstruction in the post-chemoradiotherapy failures, is often associated with poor functional outcomes, poor HRQoL and limited cure rates.
  *Drivers for change*: Health-related quality of life; function; healthcare cost.
3. Human papilloma virus (HPV) testing: It is conceivable that it is possible to de-escalate treatment in some HPV positive patients. Similar survival outcomes may be achieved by the use of cetuximab and radiotherapy rather than platinum-based chemoradiotherapy.
  *Drivers for change*: Health-related quality of life.

### Larynx

1. Early stage disease: Laser excision rather than primary radiotherapy for suitable lesions.
  *Drivers for change*: Patient choice based on equivalent HRQoL and survival.
2. Advanced stage disease: There is debate about chemoradiotherapy or laryngectomy. Following chemoradiotherapy the success and impact of laryngectomy for salvage remains to be fully determined.
  *Drivers for change*: Health-related quality of life, survival.

### Oral cavity

1. Early stage disease: There is a rationale towards primary surgery without free tissue reconstruction accepting close margins with low risk of local recurrence
  *Drivers for change*: Health-related quality of life, survival, function, cost of overall treatment.
2. Advanced stage disease: Primary surgery with free tissue reconstruction as required. However, there is discussion around the benefit of adjuvant radiotherapy.
  *Drivers for change*: Health-related quality of life, survival.

## Conclusion

The place of HRQoL assessment in head and neck cancer practice has become more defined in the last decade. It has had a major role in helping to shape treatment strategies and patient support. More evidence is yet to emerge to improve guidance as to how to use HRQoL at an individual patient level and also reflect the trade off between marginal survival improvements and increased treatment burden and poorer HRQoL. Advances in information technology will make it easier for HRQoL to assist in decision making, delivery of information, identification of problem areas, the identification of risk groups, and to drive support and interventions aimed at improving the HRQoL outcomes.

**Recommendations**

• Health-related quality of life is integral to treatment planning, refining treatment protocols, and more personalised follow-up support (G)
• Health-related quality of life and patient concerns should be regularly assessed during patient care (G)
• Health-related quality of life assessment and patient concerns on an individual patient basis can be helpful to trigger multi-professional support and interventions (G)

### Directions for the future

1. Holistic assessment integrated into clinical practice and patient reported outcomes reported in national datasets.
2. Survivorship issues addressed through interventions and empowering patients to develop skills and confidence for self-management.
3. Evidence base related to interventions, e.g. AFTER intervention for fear of recurrence.
4. A better understanding of late effects of treatment.
5. Partnership and marital issues are no doubt of significant importance, as well as grandparents and children (family). Interventions need to include couple therapy and family therapy and practitioners need to be trained in these approaches as well as individual counselling etc.
6. Wider use of information technology to allow HRQoL and patient concerns to be more readily available in clinics and across the multi-professional team.
